# Use of hormonal contraceptives and antidepressants and risks of suicidal behavior and accidents among women with premenstrual disorders: a nationwide cohort study

**DOI:** 10.1186/s12916-022-02671-z

**Published:** 2022-12-15

**Authors:** Qian Yang, Tyra Lagerberg, Arvid Sjölander, Elizabeth R. Bertone-Johnson, Fang Fang, Weimin Ye, Zheng Chang, Unnur A. Valdimarsdóttir, Donghao Lu

**Affiliations:** 1 Institute of Environmental Medicine, Karolinska Institutet, Nobels Väg 12A, 171 77 Stockholm, Sweden; 2 Department of Medical Epidemiology and Biostatistics, Karolinska Institutet, 171 77 Stockholm, Sweden; 3 Department of Biostatistics and Epidemiology, School of Public Health and Health Sciences, University of Massachusetts Amherst, Amherst, MA-01003 USA; 4 Department of Health Promotion and Policy, School of Public Health and Health Sciences, University of Massachusetts Amherst, Amherst, MA-01003 USA; 5 Center of Public Health Sciences, Faculty of Medicine, University of Iceland, IS-101 Reykjavík, Iceland; 6 Department of Epidemiology, Harvard TH Chan School of Public Health, Boston, MA-02115 USA

**Keywords:** Premenstrual disorders, Hormonal contraceptives, Antidepressants, Suicidal behavior, Accidents, Cohort study

## Abstract

**Background:**

Women with premenstrual disorders (PMDs) are at increased risks of suicidal behavior and accidents. However, the effect of PMD first-line treatment on such risks have not been assessed.

**Methods:**

To study the association between use of hormonal contraceptives or antidepressants and subsequent risks of suicidal behavior and accidents among women with PMDs. We conducted a nationwide register-based cohort study with between- and within-individual analyses in Sweden. All women with a clinical diagnosis/indication of PMDs recorded in the Patient Register and the Prescribed Drug Register during 1987–2011 were included (*n* = 23 029, age 15–52 years). Information on hormonal contraceptives and antidepressants prescribed for these women was obtained from the Prescribed Drug Register. Events of suicidal behavior (complete suicide and suicide attempt) and accidents were separately identified through the Patient and the Causes of Death Registers. Incidence rate ratios (IRRs) and 95% confidence intervals (CIs) of suicidal behavior and accidents after use of hormonal contraceptives or antidepressants were estimated in between-individual and within-individual analyses (i.e., comparing the risk between use and no use in the same individual) using Poisson regression.

**Results:**

Women with PMDs were followed for a median of 6.2 years. Compared to no use of hormonal contraceptives, use of hormonal contraceptives was associated with a lower risk of suicidal behavior in both between-individual (IRR 0.76, 0.43–1.34) and within-individual analyses (IRR 0.65, 0.51–0.83). These risk reductions were primarily restricted to combined products (IRR 0.18, 0.07–0.47 and 0.19, 0.08–0.42 in between- and within-individual analyses) and observed among women with/without psychiatric comorbidities (*p* for interaction 0.830 and 0.043 in between- and within-individual analyses). Yet, the use of hormonal contraceptives was not consistently associated with risk of accidents between between-individual (IRR 1.13, 1.01–1.27) and within-individual analyses (IRR 1.01, 0.92–1.11). Use of antidepressants was associated with a higher risk of suicidal behavior and accidents in both between- and within-individual analyses.

**Conclusions:**

Our findings suggest that use of hormonal contraceptives, particularly combined products, is associated with reduced rates of suicidal behaviors, but not accidents, among women with PMDs. The estimates for antidepressants may be biased by indication.

**Supplementary information:**

The online version contains supplementary material available at 10.1186/s12916-022-02671-z.

## Background

Premenstrual disorders (PMDs) are characterized by a range of psychological and physical symptoms that manifest 1–2 weeks before menstruation and significantly resolve after the menstruation begins [1]. PMDs primarily encompass premenstrual syndrome (PMS) and premenstrual dysphoric disorder (PMDD) [2]; the latter is characterized by more severe affective symptoms and functional impairment [3]. The prevalence is reported to be 20–40% for PMS and 2–8% for PMDD [1]. The chronicity and cyclicity of premenstrual symptoms may impose substantial functional impairment among affected women; for PMDD, it is suggested to be comparable to that of major depression [4]. Emerging evidence indicates that PMDs may also have long-lasting impact on major health outcomes. A recent study from our group showed that, compared to women without PMDs, women with PMDs have a more than doubled risk of suicidal behavior and a more than 30% increased risk of accidents [5]. This highlights the importance of preventing such devastating consequences with clinical management.

Hormonal contraceptives and antidepressants are recommended as first-line treatments for PMDs [2]. However, 10–40% of PMDs do not respond to selective serotonin reuptake inhibitors (SSRI) [6] or combined oral contraceptive (COC) containing drospirenone [7]. Even for patients that respond to these treatments, impairments in interpersonal relationships may still predispose these women to increased risks of suicidal behavior [8] and accidents [9]. Moreover, PMDs are highly comorbid with depression and anxiety, [10] which are strong predictors of suicidal behavior [11]. It remains unknown if current treatments for PMDs can adequately manage these comorbidities. Previous randomized controlled trials have primarily focused on symptom relief [12–15], whereas rare outcomes such as suicidal behavior have been difficult to study. To our best knowledge, no study has investigated whether and which treatment of PMDs is associated with reduced risks of suicidal behavior and accidents among affected women.

Leveraging a national cohort of all women with clinical indications of PMDs in Sweden, we assessed the association between the use of hormonal contraceptives/antidepressants and risks of suicidal behavior and accidents, using both between- and within-individual analyses; the latter was primarily used to adjust for unmeasured confounders that are stable over time (e.g., indication bias due to genetic factors that influence the severity of PMDs or psychiatric/gynecological comorbidities).

## Methods

### Study population

Using the Swedish national registers, we conducted a nationwide registry-based cohort study of women born during 1960–1995 and with a clinical diagnosis/indication of PMDs recorded in the Patient Register (NPR) and the Prescribed Drug Register during 1987–2011 (*N* = 23,367). Prospective symptom rating for two menstrual cycles are required for PMD diagnosis according to the Swedish clinical guidelines [16]. As previously described, [5] we first identified clinical diagnoses of PMDs from NPR using ICD codes (625E in ICD-9 and N943 in ICD-10). NPR has nationwide coverage on inpatient care since 1987 and more than 80% coverage of specialist-based outpatient visits since 2001 and has high validity (positive predicted value of 85–95% across diseases) [17]. However, the diagnoses made in primary care are not covered in NPR, and around half of the psychiatric conditions are handled in primary care in Sweden [18]. The Prescribed Drug Register covers information on all drugs prescribed in both primary and secondary care and dispensations from all pharmacies in Sweden from July 2005 onward [19]. Therefore, we also identified PMDs by searching the Prescribed Drug Register for any PMD diagnosis or explicit indication of PMD treatment in prescriptions of antidepressants (ATC code: N06AB, N06AX, N06AA) and hormonal contraceptives (G03A, G02B) [5]. Information collected in the Register includes drug identity, package size, number of packages dispensed, dates of dispensation, and a free-text variable that includes treatment indication and/or instructions from the prescriber. The study population was defined as women with clinical indications of PMDs, referred to as PMD patients for brevity.

Over 70% of the PMDs patients have symptom onset in adolescence; [20] yet, it often takes years and many healthcare visits to receive a diagnosis, usually occurring in the 30s [21]. Therefore, we followed all women from July 1, 2005, or their 15th birthday (as 96% of Swedish women had menarche by age 15), [22] whichever occurred last, until death, emigration, bilateral oophorectomy, or hysterectomy, July 31, 2011, or their 52nd birthday (the mean age of menopause among Swedish women), [23] whichever occurred first, through cross-linkage to the Causes of Death and the Migration Registers. We excluded 338 women who had bilateral oophorectomy or hysterectomy (*n* = 197), emigrated permanently (*n* = 134), or died (*n* = 7) before cohort entry, leaving 23,029 PMDs patients in analysis.

### Use of antidepressants and hormonal contraceptives

All prescriptions for antidepressants (N06AB, N06AX, N06AA) and hormonal contraceptives for systemic use (G03A except G03AD for emergency contraceptives) were identified from the Prescribed Drug Register, regardless of the actual indication for the prescription. For instance, antidepressants may be prescribed for depression, while hormonal contraceptives might be used for contraception purpose. Prescriptions that were returned to pharmacies after dispensation were excluded. The duration of dispensed medication was estimated using the prescribed total amount and the estimated daily dosage. The Defined Daily Dose was used as the daily dosage for contraceptives. For antidepressants, a newly developed algorithm which uses machine-learning was used to predict the clinician-prescribed daily dosage from free-text prescription (where prescribers indicate the daily dosage and whether dosage changes over time) [24]. The algorithm considers stepwise dose change (titration), which is common at initiation of antidepressant use. The overall accuracy of the daily dosage prediction was estimated to be 97%, [25] which was confirmed in our data by manually checking a random sample of 100 prescriptions.

The use of medications was treated as a time-varying exposure. Discontinuation was considered upon dispensation of another medication of the same type (antidepressant or hormonal contraceptive) unless the same medication was redeemed within 30 days. For subgroup analyses, hormonal contraceptives were classified into combined products (G03AA and G03AB) and progestin-only products (G03AC); antidepressants were classified into selective serotonin reuptake inhibitors (SSRIs, N06AB), non-selective monoamine reuptake inhibitors (NSMRIs, N06AA), and other antidepressants (N06AX).

### Ascertainment of suicidal behavior and accidents

We identified all events of suicidal behavior (including completed suicide and suicide attempt) and accidents that resulted in a hospital visit (as the primary or a secondary diagnosis) or death (as the underlying or a contributory cause), during the follow-up period through cross-linkage to NPR and the Causes of Death Register as described previously (suicidal behavior: X60-X84, Y870 in ICD10; accidents: V01-X59, Y85-Y86 in ICD10) [26]. NPR has a positive predicted value of 95% for suicidal behavior and accidents [17]. Causes of deaths, including suicide and accidents, recorded in the Causes of Death Register are highly accurate and complete [27, 28].

### Covariates

Information on country of birth, year of birth, and region of residence was obtained from the Swedish Population and Housing Census in 1990. Participants’ highest educational level was retrieved from the Swedish Education Register, with the latest update in 2001. Comorbid psychiatric diagnoses were identified from NPR from 1981 onward (290–319 in ICD-8/9, F10-F90 in ICD-10) and were treated as a time-varying covariate. The covariates were categorized as shown in Table 1.

**Table 1:** Characteristics of women with premenstrual disorders (PMDs) in relation to use of antidepressants and hormonal contraceptives

	**Hormonal contraceptives**		**Antidepressants**	
	**No use**	**Use**	**No use**	**Use**
**Women, *N***^**a**^	23,013	10,858	23,025	17,863
**Follow-up periods, *N***	134,530	21,605	107,709	48,426
**Person-years**	122,114	16,832	108,110	30,836
	**Mean [SD]**	**Mean [SD]**	**Mean [SD]**	**Mean [SD]**
**Age at follow-up**	36.9 [7.6]	33.5 [8.3]	35.7 [8.0]	38.0 [7.1]
**Duration of use, days**	342.0 [479.0]	219.7 [292.3]	379.6 [514.7]	203.6 [265.9]
	***N* (%)**	***N* (%)**	***N* (%)**	***N* (%)**
**Country of birth**				
Sweden	117,301 (87.2)	19,161 (88.7)	94,253 (87.5)	42,209 (87.2)
Others	17,229 (12.8)	2444 (11.3)	13,456 (12.5)	6217 (12.8)
**Year of birth**				
1960–1964	30,428 (22.6)	2676 (12.4)	21,147 (19.6)	11,957 (24.7)
1965–1969	36,798 (27.4)	4382 (20.3)	27,181 (25.2)	13,999 (28.9)
1970–1974	29,799 (22.2)	4778 (22.1)	23,832 (22.1)	10,745 (22.2)
1975–1979	17,787 (13.2)	3663 (17.0)	15,436 (14.3)	6014 (12.4)
1980–1984	11,351 (8.4)	3048 (14.1)	10,906 (10.1)	3493 (7.2)
1985–1990	6892 (5.1)	2429 (11.2)	7470 (6.9)	1851 (3.8)
**Educational level**				
Primary	17,718 (13.2)	3329 (15.4)	14,871 (13.8)	6176 (12.8)
High school	65,112 (48.4)	9734 (45.1)	51,067 (47.4)	23,779 (49.1)
College and beyond	43,454 (32.3)	5728 (26.5)	32,969 (30.6)	16,213 (33.5)
Unknown	8246 (6.1)	2814 (13.0)	8802 (8.2)	2258 (4.7)
**Region of residency**				
South	23,401 (17.4)	4077 (18.9)	19,244 (17.9)	8234 (17.0)
Middle	77,359 (57.5)	11,898 (55.1)	61,100 (56.7)	28,157 (58.1)
North	24,341 (18.1)	3807 (17.6)	19,256 (17.9)	8892 (18.4)
Unknown	9429 (7.0)	1823 (8.4)	8109 (7.5)	3143 (6.5)
	**PYs (%)**	**PYs (%)**	**PYs (%)**	**PYs (%)**
**Psychiatric comorbidities**				
No	108,580 (88.9)	14,960 (88.9)	98,526 (91.1)	25,014 (81.1)
Yes	13,534 (11.1)	1872 (11.1)	9584 (8.9)	5822 (18.9)

*N*, number; *PYs*, person years; *SD*, standard deviation

^a^One individual could contribute to multiple follow-up periods and medication groups

### Statistical analysis

Individuals contributed person-time to use or no use of hormonal contraceptive/antidepressant and could have multiple events of suicidal behavior or accidents during follow-up. First, we calculated unadjusted incidence rates (IR; number of events divided by accumulated person-years) of suicidal behavior and accidents in all groups. We then used Poisson regression to estimate incidence rate ratios (IRRs) [29–32] and 95% confidence intervals (CIs) of outcomes by comparing use of hormonal contraceptives/antidepressants with no use between individuals (person-time as offset) [33]. We also performed within-individual analysis by contrasting the rates within each individual discordant on medication status using conditional Poisson regression. This analysis inherently controls for factors that are constant within each individual during the follow-up [34, 35].

Use of the other medication was mutually adjusted for in a time-varying way in all analyses. Country of birth, age (time varying every year), educational level, region of residency, and psychiatric comorbidities were additionally adjusted in between-individual analysis. Non-independence of records contributed by a same individual was corrected for with a robust sandwich estimator of variance [36]. In the within-individual analysis, age and psychiatric comorbidities were additionally adjusted for.

A series of sensitivity analyses were conducted to assess the robustness of our main findings. To assess potential differences among PMD patients identified through clinical diagnoses vs. treatment indications, we performed analyses restricted to patients identified from the Patient and the Prescribed Drug Registers, separately. To alleviate the concern of PMD diagnosis validity, we restricted the analysis to PMD patients with at least two consecutive specialists-made diagnoses at least 28 days apart. To avoid potential dependence between repeated events, we performed analyses using the first event in each use/non-use period. To assess the influence of differential risks before and after PMD diagnosis, [5] we repeated the analysis by excluding person-times before PMD diagnosis. Finally, to assess the delayed medication effect, we excluded the first 28 days since the prescription.

Based on findings from the main analysis, we only focused on suicidal behavior in subsequent analyses. To provide insights into different types of medication, we performed subgroup analyses by different types of hormonal contraceptives and antidepressants. PMD is often comorbid with depression/anxiety, [1] which are independent indications for antidepressants [37] and are associated with increased risks of suicidal behavior [11]. We therefore performed stratified analysis by psychiatric comorbidities to illustrate the potential risk modification.

We also performed several additional analyses to better understand the association between use of hormonal contraceptives and suicidal behavior. To explore the duration effect, we estimated IRRs of suicidal behavior across different time windows (0–3, 4–6, 7–12, and > 12 months) in relation to use of contraceptives. Moreover, use of contraceptives is related to relationship status, which might modify the risk of suicidal behavior. Due to the lack of information on dynamic changes of relationship status over the follow-up, we conducted stratified analyses by age at medication and parity at baseline as proxies for relationship status.

Data were prepared in the SAS statistical software, version 9.4 (SAS Institute). The core processes of the algorithm for antidepressants were carried out in Python (version 3.7.1) using Keras [38]. Data analysis was done in Stata 17 (STATA). The statistical significance was set at the nominal two-sided 5% level.

## Results

### Characteristics

The study included 23,029 women with PMDs with mean age of 35.6 (standard deviation, SD 7.4 years) at PMDs diagnosis and mean age of 34.2 years (SD 7.6 years) at cohort entry. Compared to no use, use of hormonal contraceptives was more common at younger age and thereby also at lower educational level (Table 1). By contrast, use of antidepressants was more common at older age and at higher educational level compared to no use.

### Risk of suicidal behavior and accidents

During a median follow-up of 6.2 years over 138,946 person-years, we identified a total of 932 events of suicidal behavior and 6 875 accidents. Compared to no use, use of hormonal contraceptives was associated with a lower risk of suicidal behavior in both between- and within-individual analyses (adjusted IRR 0.76, 95% CI 0.43–1.34; IRR 0.65, 0.51–0.83 respectively; Table 2). Use of contraceptives was associated with a slightly higher risk of accidents in between-individual analysis (IRR 1.13, 1.01–1.27), but not in within-individual analysis (IRR 1.01, 0.92–1.11). Use of antidepressants was associated with an increased risk of suicidal behavior (IRR 3.06, 2.18–4.30 in between-individual analysis and IRR 1.97, 1.66–2.34 in within-individual analysis) and accidents (IRR 1.15, 1.05–1.25 in between-individual analysis and IRR 1.17, 1.09–1.26 in within-individual analysis) compared to no use periods.

**Table 2:** Associations of use of antidepressants and hormonal contraceptives with subsequent risks of suicidal behavior and accidents among women with premenstrual disorders (PMDs)

	**Events**	**Between-individual analysis**		**Within-individual analysis**	
	*N* (IR)	IRR (95% CIs)^**a**^	IRR (95% CIs)^b^	IRR (95% CIs)^**c**^	IRR (95% CIs)^d^
***Suicidal behavior***					
**Hormonal contraceptives**					
No use	809 (6.6)	Ref	Ref	Ref	Ref
Use	123 (7.3)	1.09 (0.67–1.76)	0.76 (0.43–1.34)	0.65 (0.51–0.83)	0.65 (0.51–0.83)
**Antidepressants**					
No use	380 (3.5)	Ref	Ref	Ref	Ref
Use	552 (17.9)	5.09 (3.77–6.88)	3.06 (2.18–4.30)	1.86 (1.57–2.20)	1.97 (1.66–2.34)
***Accidents***					
**Hormonal contraceptives**					
No use	5,968 (48.9)	Ref	Ref	Ref	Ref
Use	907 (53.9)	1.10 (0.99–1.23)	1.13 (1.01–1.27)	1.01 (0.92–1.11)	1.01 (0.92–1.11)
**Antidepressants**					
No use	5,047 (46.7)	Ref	Ref	Ref	Ref
Use	1,828 (59.3)	1.27 (1.17–1.38)	1.15 (1.05–1.25)	1.13 (1.05–1.21)	1.17 (1.09–1.26)

*CI*, confidence interval; *IR*, crude incidence rate per 1000 person-years; *IRR*, incidence rate ratio; *N*, number

^a^Use of hormonal contraceptives and antidepressants were mutually adjusted for and the analysis was accounted for non-independence of follow-ups contributed by a same individual using robust sandwich estimator of variance

^b^Estimates were additionally adjusted for age at follow-up, educational level (primary school, high school, or college and beyond), country of birth (Sweden or other), region of residency (south, middle, or north of Sweden), and psychiatric comorbidities (yes or no)

^c^Use of hormonal contraceptives and antidepressants were mutually adjusted for and the analysis was conditioned on each individual

^d^Estimates were additionally adjusted for age at follow-up and psychiatric comorbidities (yes or no)

Similar results were found when we studied PMDs patients ascertained from the Patient and the Prescribed Drug Registers separately; restricted to PMDs patients with at least two consecutive specialist-made diagnoses 28 days apart; examined the risk of the first event of outcomes; excluded the person-time before PMDs diagnosis; and introduced a 28-day lag time for the medications (Additional file 1: Table S1-S4). Given the null association between studied medications and accidents, we primarily focused on suicidal behavior in subsequent analyses.

### Type-specific medication

Lower risk of suicidal behavior when using contraceptives was confined to combined products (IRR 0.18, 0.07–0.47 in between-individual analysis and 0.19, 0.08–0.42 in within-individual analysis) but not progestin-only products. Higher risk of suicidal behavior was suggested for all types of antidepressants, albeit not statistically significant for NSMRIs. The higher risk of suicidal behavior was noted particularly for other types of antidepressants such as mirtazapine and duloxetine (Table 3).

**Table 3:** Associations of type-specific use of hormonal contraceptives and antidepressants with subsequent risk of suicidal behavior among women with premenstrual disorders (PMDs)

		**Between-individual analysis**	**Within-individual analysis**
	***N* (IR)**	**IRR (95% CIs)**^**a**^	**IRR (95% CIs)**^**b**^
*Hormonal contraceptives*			
No use	809 (6.6)	Ref	Ref
Combined	56 (6.3)	0.18 (0.07–0.47)	0.19 (0.08–0.42)
Progestin-only	67 (8.4)	0.86 (0.28–2.58)	1.04 (0.61–1.76)
*Antidepressants*			
No use	380 (3.5)	Ref	Ref
SSRIs	299 (12.1)	2.22 (1.52–3.24)	1.80 (1.45–2.25)
NSMRIs	18 (10.7)	1.21 (0.61–2.39)	1.83 (0.68–4.93)
Others	235 (53.6)	5.30 (3.33–8.45)	2.16 (1.66–2.82)

*CI*, confidence interval; *IR*, crude incidence rate per 1000 person-years; *IRR*, incidence rate ratio; *N*, number

^a^Use of hormonal contraceptives and antidepressants were mutually adjusted for and the analysis was accounted for non-independence of follow-ups contributed by a same individual using robust sandwich estimator of variance. Estimates were also adjusted for age at follow-up, educational level (primary school, high school, or college and beyond), country of birth (Sweden or other), region of residency (south, middle, or north of Sweden), and psychiatric comorbidities (yes or no)

^b^Use of hormonal contraceptives and antidepressants were mutually adjusted for and the analysis was conditioned on each individual. Estimates were also adjusted for age at follow-up and psychiatric comorbidities (yes or no)

### Effect modification by psychiatric comorbidities

The lower risk of suicidal behavior with use of contraceptives was noted among PMDs patients with/without psychiatric comorbidities in the within-individual analysis (*p* for interaction 0.043, Table 4), although lack of statistical significance was observed in the between-individual analysis (*p* for interaction 0.830, Table 4). The higher risk of suicidal behavior when using antidepressants was evident regardless of psychiatric comorbidities, although the magnitude was greater among women without psychiatric comorbidities (*p* for interaction 0.048 and 0.030 in between- and within-individual analyses).

**Table 4:** Associations of use of hormonal contraceptives and antidepressants with subsequent risk of suicidal behavior among women with premenstrual disorders (PMDs), stratified by psychiatric comorbidities

	**Events**	**Between-individual analysis**	**Within-individual analysis**
	*N* (IR)	IRR (95% CIs)^a^	IRR (95% CIs)^b^
**Hormonal contraceptives**			
***Without psychiatric comorbidities***			
No use	161 (1.5)	Ref	Ref
Use	24 (1.6)	0.70 (0.40–1.24)	0.40 (0.24–0.69)
***With psychiatric comorbidities***			
No use	648 (47.9)	Ref	Ref
Use	99 (52.9)	0.77 (0.39–1.50)	0.74 (0.56–0.98)
***P for interaction***		0.830	0.043
**Antidepressants**			
***Without psychiatric comorbidities***			
No use	91 (0.9)	Ref	Ref
Use	94 (3.8)	4.66 (3.29–6.62)	2.76 (1.95–3.93)
***With psychiatric comorbidities***			
No use	289 (30.2)	Ref	Ref
Use	458 (78.7)	2.78 (1.89–4.09)	1.79 (1.48–2.16)
***P for interaction***		0.048	0.030

*CI*, confidence interval; *IR*, crude incidence rate per 1 000 person-years; *IRR*, incidence rate ratio; *N*, number

^a^Use of hormonal contraceptives and antidepressants were mutually adjusted for and the analysis was accounted for non-independence of follow-ups contributed by a same individual using robust sandwich estimator of variance. Estimates were also adjusted for age at follow-up, educational level (primary school, high school, or college and beyond), country of birth (Sweden or other), region of residency (south, middle, or north of Sweden), and psychiatric comorbidities (yes or no)

^b^Use of hormonal contraceptives and antidepressants were mutually adjusted for and the analysis was conditioned on each individual. Estimates were also adjusted for age at follow-up and psychiatric comorbidities (yes or no)

### Additional analyses for hormonal contraceptives

**Fig. 1:**
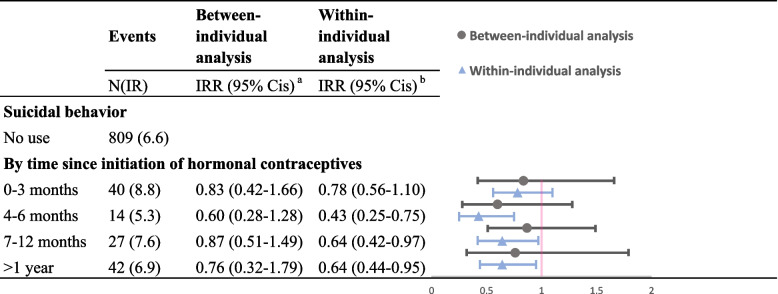
Use of hormonal contraceptives and subsequent risk of suicidal behavior among women with premenstrual disorders (PMDs), by time since the prescription. Superscript small letter “a” (^a^) indicates the following: use of hormonal contraceptives and antidepressants were mutually adjusted for and the analysis was accounted for non-independence of follow-ups contributed by a same individual using robust sandwich estimator of variance. Estimates were also adjusted for age at follow-up, educational level (primary school, high school, or college and beyond), country of birth (Sweden or other), region of residency (south, middle, or north of Sweden), and psychiatric comorbidities (yes or no). Superscript small letter “b” (^b^) indicates the following: use of hormonal contraceptives and antidepressants were mutually adjusted for and the analysis was conditioned on each individual. Estimates were also adjusted for age at follow-up and psychiatric comorbidities (yes or no)

The lower risk of suicidal behavior with contraceptives use among women with PMDs was not statistically significant within the first three months in a prescribed period in between- and within-individual analyses (Fig. 1). Such association became significant from three months onwards in the within-individual analysis. Finally, IRRs of suicidal behavior when using contraceptives were largely comparable between age (i.e., ≤ 30 or > 30 years at medication) and parity groups (i.e., with or without a child at baseline) (Additional file 1: Table S5).

## Discussion

Accumulating evidence suggests a higher risk of suicidal behavior and accidents among PMD patients, [5, 39] whereas existing studies examining the effect of hormonal contraceptives or antidepressants used among women with PMDs primarily focus on symptom relief [6, 7, 12–15]. To date, our study is the first to examine the effect of hormonal contraceptives and antidepressants on suicidal behavior and accident among women with PMDs. In this nationwide cohort of 23,029 women with a clinical indication of PMDs, we found a statistically significant lower risk of suicidal behavior when using contraceptives of combined products, compared to no-use periods, in both between- and within-individual analyses. Such risk reduction was evident regardless of psychiatric comorbidities and was most pronounced after 3 months since the prescription claim. On the other hand, antidepressant use was associated with a higher risk of suicidal behavior, particularly for types other than SSRIs (e.g., tricyclic antidepressants), which are often prescribed for patients with more severe symptomology and not responsive to SSRIs, [40] suggesting a strong indication bias.

Hormonal contraceptives may reduce premenstrual symptoms and psychosocial impairment through regulating the hypothalamic pituitary adrenal (HPA) axis and modulating the neurotransmitter pathway [7, 14, 15]. Such improvement might lead to a decreased risk of suicidal behavior given its sociopsychological characteristics. Indeed, our study on PMD patients highlights a lower risk of suicidal behavior when using contraceptives, suggesting a potential benefit of contraceptive use from risk reduction in suicidal behavior among patients. Moreover, previous studies support the efficacy of combined oral contraceptives in symptom relief for PMDs, whereas the findings on progestin-only products are inconsistent [41]. In line with this, we observed a lower risk of suicidal behavior when using combined products but not progestin-only products. This might be due to the synergistic effect of estrogen and progesterone, i.e., the combination produces a greater effect than the addictive effect in many biological processes such as the serotonin receptor binding potential [42]. Worth noting is that, compared to those who were not prescribed with contraceptives, patients with prescribed contraceptives may have more severe symptoms or comorbid with gynecological conditions (e.g., endometriosis), who are at increased risk of suicidal behavior and accidents [43, 44]. Interestingly, the positive association between use of hormonal contraceptives and accidents in between-individual analysis attenuated to null in within-individual analysis, supporting the advantage of this design in addressing time-stable unmeasured confounders. By contrast, the association with suicidal behavior was consistent between the two analyses, suggesting that this association cannot entirely be explained by time-stable unmeasured confounders such as indication bias. On the other hand, relationship status might affect the use of hormonal contraceptives and thereby confound the studied association through psychological well-being [45]. However, as two indicators of relationship status, we obtained largely similar results among women under and above age 30 and among women with and without a child. Nevertheless, future studies with information on relationship status are needed.

Antidepressants are also the first-line treatment for PMDs [2]. Specifically, SSRIs were reported to have an efficacy of 60–70% in symptom mitigation, compared with a 30% response rate for placebo [46]. However, we observed that use of antidepressants was associated with higher risk of suicidal behavior among patients with PMDs, which might be due to indication bias. Around 40% of PMDs patients suffer comorbid depressive symptoms, [10] and those patients are more likely to be prescribed antidepressants. Those psychiatric comorbidities are strong indicators of suicidal behavior [11]. Indeed, the association between use of antidepressants and suicidal behavior was significantly attenuated in the within-individual analysis, supporting a strong indication bias. Moreover, the association was most evident for antidepressants other than SSRIs, which are more commonly prescribed for patients with SSRI-resistant depression or panic attack [40]. Additionally, we lacked data on psychiatric symptoms handled in primary care, which accounts for around half of all psychiatric conditions in Sweden [18]. Therefore, the greater association observed among patients without psychiatric comorbidities was likely because we missed indication of psychiatric disorders diagnosed in primary care. Taken together, the observed association between antidepressant use and suicidal behavior among PMD patients is likely inflated by indication bias.

The major strength of the study is the nationwide prospective cohort design with complete follow-up. The within-individual comparison largely controls for unmeasured time-stable confounding, including indication bias from factors that are stable within individuals over the study period. For instance, genetic factors have been suggested to play a crucial role in the onset and progression of psychiatric disorders [47] as well as sensitivity to treatment [48]. These genetic factors could therefore confound the studied association in the between-individual analysis but were inherently controlled for in the within-individual analysis. However, there are several limitations. Firstly, the diagnoses of PMDs have not been specifically validated in NPR [3]. However, NPR has high validity in general (positive predicted value 85–95%) [49] and for a wide range of psychiatric disorders [50–53] and gynecological diseases [17, 54]. Daily prospective evaluation for at least two menstrual cycles is also required for PMD diagnosis according to the Swedish clinical guidelines in many regions, which is often well followed in the tax-funded healthcare system [16]. Moreover, our sensitivity analysis restricted to PMDs with two consecutive diagnoses (≥ 28 days apart) made by specialists, presumably of high specificity, yielded similar results. Secondly, we cannot guarantee intake of the dispensed medication. However, PMD patients with worsening symptoms and thereby at higher risk for suicidal behavior are more likely to discontinue use of contraceptives [55]. This misclassification would have attenuated the association between use of contraceptives and suicidal behavior. Similarly, we were not able to identify other suicidal behaviors that did not result in hospital visit, such as suicidal ideation. In addition, most of women with a suicidal behavior identified had suicide attempt (i.e., only 13 events were completed suicide), and we did not have statistical power to investigate whether the association for completed suicide would differ from that of suicide attempt. Future studies with larger sample sizes or longer follow-up are needed to study subtypes of suicidal behaviors in details. Third, unmeasured or time-varying confounders (e.g., severity of psychiatric comorbidities and relationship status) were not addressed, and we cannot make causal interpretations from the results. Fourth, we lacked statistical power to explore the combined use of hormonal contraceptives and antidepressants. Moreover, PMDs diagnosed only in primary care and women not treated for their PMDs were not included in our analysis. These patients likely had milder symptomology and lower risk of suicidal behavior compared to included individuals. The benefit of hormonal contraceptives on suicidal behavior risk is unknown for mild PMDs. Lastly, future large-scale cohort studies and clinical trials across countries, cultures, and ethnicities are needed to validate our results and evaluate the generalizability of the findings.

## Conclusions

Our findings suggest that use of hormonal contraceptives may be associated with a lower risk of suicidal behavior among women with PMDs, particularly for combined products. If confirmed in future clinical trials, the use of combined contraceptives may help mitigate risk of suicidal behaviors among women with PMDs.

## Supplementary information

**Additional file 1.** Description of data (reference: https://bmcmedicine.biomedcentral.com/submission-guidelines/preparing-your-manuscript#preparing+additional+files).

## Data Availability

Data are from the Swedish Population and Housing Census, Causes of Death Register, Migration Register, Patient Register, Prescribed Drug Register, and Swedish Education Register. According to the Swedish law, data cannot be put into a public data repository but are available by applying through Statistics Sweden or the Swedish National Board of Health and Welfare. Detailed information on data application can be found in their official sites: https://www.scb.se/vara-tjanster/bestalla-mikrodata/ and https://bestalladata.socialstyrelsen.se/.

## Abbreviations

PMDs: Premenstrual disorders
IRRs: Incidence rate ratios
CIs: Confidence intervals
PMS: Premenstrual syndrome
PMDD: Premenstrual dysphoric disorder
SSRI: Selective serotonin reuptake inhibitors
COC: Combined oral contraceptive
NPR: Patient Register
HPA: Hypothalamic pituitary adrenal
