# Supplementary material for: Use of hormonal contraceptives and antidepressants and risks of suicidal behavior and accidents among women with premenstrual disorders: a nationwide cohort study
Source: BMC Med. 2022 Dec 15;20:482. doi: 10.1186/s12916-022-02671-z (PMC9753283; doi:10.1186/s12916-022-02671-z)
Supplement: Supplementary file 1 — Additional file 1. Description of data (reference: https://bmcmedicine.biomedcentral.com/submission-guidelines/preparing-your-manuscript#preparing+additional+files). [file 12916_2022_2671_MOESM1_ESM.docx]

**Table S1. Associations of use of antidepressants and hormonal contraceptives with subsequent risks of suicidal behavior and accidents among women with premenstrual disorders (PMDs) identified through different approaches.**

|  |  | **Population analysis** | | **Within-individual analysis** | |
| --- | --- | --- | --- | --- | --- |
|  | N (IR) | IRR (95% CIs) **^a^** | IRR (95% CIs) ^b^ | IRR (95% CIs) **^c^** | IRR (95% CIs) ^d^ |
| ***Restricted to specialists diagnosed PMDs identified from the Patient Register*** | | | | | |
| ***Suicidal behavior*** | |  |  |  |  |
| **Hormonal contraceptives** |  |  |  |  |  |
| No use | 478 (8.1) | Ref. | Ref. | Ref. | Ref. |
| Use | 91 (11.0) | 1.30 (0.70-2.43) | 1.00 (0.50-2.00) | 0.75 (0.56-1.02) | 0.72 (0.53-0.98) |
| **Antidepressants** |  |  |  |  |  |
| No use | 230 (4.3) | Ref. | Ref. | Ref. | Ref. |
| Use | 339 (24.6) | 5.70 (3.72-8.74) | 3.17 (1.94-5.17) | 2.08 (1.66-2.60) | 2.08 (1.66-2.61) |
| ***Accidents*** |  |  |  |  |  |
| **Hormonal contraceptives** |  |  |  |  |  |
| No use | 3,312 (56.3) | Ref. | Ref. | Ref. | Ref. |
| Use | 508 (61.3) | 1.09 (0.93-1.26) | 1.10 (0.93-1.30) | 1.01 (0.89-1.15) | 1.01 (0.89-1.14) |
| **Antidepressants** |  |  |  |  |  |
| No use | 2,859 (53.6) | Ref. | Ref. | Ref. | Ref. |
| Use | 961 (69.9) | 1.30 (1.16-1.47) | 1.17 (1.03-1.33) | 1.18 (1.07-1.31) | 1.21 (1.10-1.34) |
| ***Restricted to PMDs identified from the Prescribed Drug Register*** | | | | | |
| ***Suicidal behavior*** | |  |  |  |  |
| **Hormonal contraceptives** |  |  |  |  |  |
| No use | 331 (5.2) | Ref. | Ref. | Ref. | Ref. |
| Use | 32 (3.7) | 0.72 (0.43-1.19) | 0.45 (0.26-0.78) | 0.50 (0.32-0.76) | 0.55 (0.35-0.85) |
| **Antidepressants** |  |  |  |  |  |
| No use | 150 (2.7) | Ref. | Ref. | Ref. | Ref. |
| Use | 213 (12.5) | 4.55 (3.09-6.71) | 2.71 (1.71-4.31) | 1.61 (1.25-2.08) | 1.87 (1.43-2.43) |
| ***Accidents*** |  |  |  |  |  |
| **Hormonal contraceptives** |  |  |  |  |  |
| No use | 2,656 (42.0) | Ref. | Ref. | Ref. | Ref. |
| Use | 399 (46.7) | 1.11 (0.96-1.29) | 1.14 (0.97-1.33) | 1.01 (0.88-1.17) | 1.01 (0.88-1.17) |
| **Antidepressants** |  |  |  |  |  |
| No use | 2,188 (40.0) | Ref. | Ref. | Ref. | Ref. |
| Use | 867 (50.8) | 1.27 (1.13-1.43) | 1.15 (1.01-1.30) | 1.08 (0.98-1.19) | 1.13 (1.02-1.25) |
| ***Restricted to women with at least two clinical diagnoses of PMD ascertained from the Patient Register*** | | | | | |
| ***Suicidal behavior*** | |  |  |  |  |
| **Hormonal contraceptives** |  |  |  |  |  |
| No use | 108 (9.2) | Ref. | Ref. | Ref. | Ref. |
| Use | 36 (22.7) | 2.34 (0.64-8.52) | 1.97 (0.67-5.84) | 0.87 (0.52-1.48) | 0.78 (0.46-1.33) |
| **Antidepressants** |  |  |  |  |  |
| No use | 61 (6.3) | Ref. | Ref. | Ref. | Ref. |
| Use | 83 (22.8) | 3.53 (2.09-5.95) | 2.01 (1.07-3.76) | 1.78 (1.21-2.62) | 1.48 (0.98-2.25) |
| ***Accidents*** |  |  |  |  |  |
| **Hormonal contraceptives** |  |  |  |  |  |
| No use | 718 (61.5) | Ref. | Ref. | Ref. | Ref. |
| Use | 112 (70.7) | 1.14 (0.77-1.69) | 1.23 (0.82-1.85) | 1.40 (1.06-1.85) | 1.37 (1.04-1.82) |
| **Antidepressants** |  |  |  |  |  |
| No use | 567 (58.9) | Ref. | Ref. | Ref. | Ref. |
| Use | 263 (72.3) | 1.22 (0.96-1.56) | 1.12 (0.87-1.44) | 1.29 (1.06-1.56) | 1.31 (1.08-1.59) |

CI, confidence interval; IR, crude incidence rate per 1 000 person-years; IRR, incidence rate ratio; N, number.

^a.^ Use of hormonal contraceptives and antidepressants were mutually adjusted for and the analysis was accounted for non-independence of follow-ups contributed by a same individual using robust sandwich estimator of variance.

^b.^ Estimates were additionally adjusted for age at follow-up, educational level (primary school, high school, or college and beyond), country of birth (Sweden or other), region of residency (south, middle, or north of Sweden), and psychiatric comorbidities (yes or no).

^c.^ Use of hormonal contraceptives and antidepressants were mutually adjusted for and the analysis was conditioned on each individual.

^d.^ Estimates were additionally adjusted for age at follow-up and psychiatric comorbidities (yes or no).

**Table S2. Associations of use of antidepressants and hormonal contraceptives with subsequent risks of suicidal behavior and accidents among women with premenstrual disorders (PMDs): first event**

|  |  | **Population analysis** | |
| --- | --- | --- | --- |
|  | N (IR) | **IRR (95% CIs) ^a^** | **IRR (95% CIs) ^b^** |
| ***Suicidal behavior*** |  |  |  |
| **Hormonal contraceptives** |  |  |  |
| No use | 493 (4.1) | Ref. | Ref. |
| Use | 78 (4.6) | 1.13 (0.86-1.48) | 0.73 (0.53-0.99) |
| **Antidepressants** |  |  |  |
| No use | 236 (2.2) | Ref. | Ref. |
| Use | 335 (11.0) | 5.01 (4.24-5.91) | 3.41 (2.83-4.10) |
| ***Accidents*** |  |  |  |
| **Hormonal contraceptives** |  |  |  |
| No use | 3,755 (32.2) | Ref. | Ref. |
| Use | 638 (39.0) | 1.21 (1.11-1.32) | 1.14 (1.04-1.26) |
| **Antidepressants** |  |  |  |
| No use | 3,171 (30.7) | Ref. | Ref. |
| Use | 1,222 (40.8) | 1.33 (1.24-1.42) | 1.24 (1.16-1.33) |

CI, confidence interval; IR, crude incidence rate per 1 000 person-years; IRR, incidence rate ratio; N, number.

^a.^ Use of hormonal contraceptives and antidepressants were mutually adjusted for and the analysis was accounted for non-independence of follow-ups contributed by a same individual using robust sandwich estimator of variance.

^b.^ Estimates were additionally adjusted for age at follow-up, educational level (primary school, high school, or college and beyond), country of birth (Sweden or other), region of residency (south, middle, or north of Sweden), and psychiatric comorbidities (yes or no).

**Table S3. Associations of use of antidepressants and hormonal contraceptives with subsequent risks of suicidal behavior and accidents among women with premenstrual disorders (PMDs): excluding follow-ups before receiving a diagnosis of PMD.**

|  |  | **Population analysis** | | **Within-individual analysis** | |
| --- | --- | --- | --- | --- | --- |
|  | N (IR) | IRR (95% CIs) **^a^** | IRR (95% CIs) ^b^ | IRR (95% CIs) **^c^** | IRR (95% CIs) ^d^ |
| ***Suicidal behavior*** |  |  |  |  |  |
| **Hormonal contraceptives** |  |  |  |  |  |
| No use | 479 (6.4) | Ref. | Ref. | Ref. | Ref. |
| Use | 91 (8.5) | 1.32 (0.71-2.46) | 0.94 (0.46-1.94) | 0.78 (0.58-1.04) | 0.75 (0.56-1.02) |
| **Antidepressants** |  |  |  |  |  |
| No use | 229 (3.8) | Ref. | Ref. | Ref. | Ref. |
| Use | 341 (13.6) | 3.61 (2.36-5.52) | 2.35 (1.48-3.72) | 1.41 (1.13-1.76) | 1.37 (1.10-1.71) |
| ***Accidents*** |  |  |  |  |  |
| **Hormonal contraceptives** |  |  |  |  |  |
| No use | 3,737 (49.7) | Ref. | Ref. | Ref. | Ref. |
| Use | 562 (52.3) | 1.05 (0.91-1.21) | 1.05 (0.91-1.22) | 0.91 (0.80-1.05) | 0.91 (0.79-1.04) |
| **Antidepressants** |  |  |  |  |  |
| No use | 2,882 (47.4) | Ref. | Ref. | Ref. | Ref. |
| Use | 1,417 (56.5) | 1.19 (1.08-1.32) | 1.10 (0.99-1.23) | 1.14 (1.04-1.25) | 1.10 (1.00-1.20) |

CI, confidence interval; IR, crude incidence rate per 1 000 person-years; IRR, incidence rate ratio; N, number.

^a.^ Use of hormonal contraceptives and antidepressants were mutually adjusted for and the analysis was accounted for non-independence of follow-ups contributed by a same individual using robust sandwich estimator of variance.

^b.^ Estimates were additionally adjusted for age at follow-up, educational level (primary school, high school, or college and beyond), country of birth (Sweden or other), region of residency (south, middle, or north of Sweden), and psychiatric comorbidities (yes or no).

^c.^ Use of hormonal contraceptives and antidepressants were mutually adjusted for and the analysis was conditioned on each individual.

^d.^ Estimates were additionally adjusted for age at follow-up and psychiatric comorbidities (yes or no).

**Table S4. Associations of use of antidepressants and hormonal contraceptives with subsequent risks of suicidal behavior and accidents among women with premenstrual disorders (PMDs): excluding the first 28 days of follow-up after the prescription.**

|  |  | **Population analysis** | | **Within-individual analysis** | |
| --- | --- | --- | --- | --- | --- |
|  | N (IR) | IRR (95% CIs) **^a^** | IRR (95% CIs) ^b^ | IRR (95% CIs) **^c^** | IRR (95% CIs) ^d^ |
| ***Suicidal behavior*** |  |  |  |  |  |
| **Hormonal contraceptives** |  |  |  |  |  |
| No use | 759 (6.4) | Ref. | Ref. | Ref. | Ref. |
| Use | 102 (6.9) | 1.05 (0.62-1.77) | 0.75 (0.41-1.37) | 0.59 (0.45-0.77) | 0.59 (0.45-0.78) |
| **Antidepressants** |  |  |  |  |  |
| No use | 373 (3.5) | Ref. | Ref. | Ref. | Ref. |
| Use | 488 (18.2) | 5.21 (3.82-7.10) | 3.07 (2.17-4.33) | 1.79 (1.50-2.14) | 1.92 (1.60-2.30) |
| ***Accidents*** |  |  |  |  |  |
| **Hormonal contraceptives** |  |  |  |  |  |
| No use | 5,775 (48.6) | Ref. | Ref. | Ref. | Ref. |
| Use | 789 (53.2) | 1.09 (0.97-1.23) | 1.12 (0.99-1.26) | 1.00 (0.90-1.11) | 0.99 (0.89-1.10) |
| **Antidepressants** |  |  |  |  |  |
| No use | 4,980 (46.6) | Ref. | Ref. | Ref. | Ref. |
| Use | 1,584 (59.1) | 1.27 (1.16-1.38) | 1.14 (1.04-1.25) | 1.14 (1.06-1.23) | 1.18 (1.10-1.28) |

CI, confidence interval; IR, crude incidence rate per 1 000 person-years; IRR, incidence rate ratio; N, number.

^a.^ Use of hormonal contraceptives and antidepressants were mutually adjusted for and the analysis was accounted for non-independence of follow-ups contributed by a same individual using robust sandwich estimator of variance.

^b.^ Estimates were additionally adjusted for age at follow-up, educational level (primary school, high school, or college and beyond), country of birth (Sweden or other), region of residency (south, middle, or north of Sweden), and psychiatric comorbidities (yes or no).

^c.^ Use of hormonal contraceptives and antidepressants were mutually adjusted for and the analysis was conditioned on each individual.

^d.^ Estimates were additionally adjusted for age at follow-up and psychiatric comorbidities (yes or no).

**Table S5. Associations of use of hormonal contraceptives with subsequent risk of suicidal behavior among women with premenstrual disorders (PMDs), stratified by age at use and parity.**

|  | **Events** | **Population analysis** | **Within-individual analysis** |
| --- | --- | --- | --- |
|  | **N (IR)** | **IRR (95% CIs) ^a^** | **IRR (95% CIs) ^b^** |
| **Suicidal behavior, by age at use** | |  |  |
| ≤30 years |  |  |  |
| No use | 369 (12.0) | Ref. | Ref. |
| Use | 100 (12.5) | 0.81 (0.41-1.62) | 0.67 (0.51-0.88) |
| >30 years |  |  |  |
| No use | 440 (4.8) | Ref. | Ref. |
| Use | 23 (2.6) | 0.54 (0.23-1.27) | 0.62 (0.37-1.04) |
| **By parity** |  |  |  |
| 0 |  |  |  |
| No use | 539 (11.2) | Ref. | Ref. |
| Use | 111 (11.5) | 0.77 (0.41-1.46) | 0.65 (0.50-0.85) |
| ≥ 1 |  |  |  |
| No use | 270 (3.6) | Ref. | Ref. |
| Use | 12 (1.7) | 0.50 (0.24-1.01) | 0.66 (0.30-1.43) |

CI, confidence interval; IR, crude incidence rate per 1 000 person-years; IRR, incidence rate ratio; N, number.

^a.^ Use of hormonal contraceptives and antidepressants were mutually adjusted for and the analysis was accounted for non-independence of follow-ups contributed by a same individual using robust sandwich estimator of variance. Estimates were also adjusted for age at follow-up, educational level (primary school, high school, or college and beyond), country of birth (Sweden or other), region of residency (south, middle, or north of Sweden), and psychiatric comorbidities (yes or no).

^b.^ Use of hormonal contraceptives and antidepressants were mutually adjusted for and the analysis was conditioned on each individual. Estimates were also adjusted for age at follow-up and psychiatric comorbidities (yes or no).
